# Arterial Stiffness and Pulse Wave Reflection in Young Adult Heterozygous Sickle Cell Carriers

**DOI:** 10.4274/Tjh.2012.0205

**Published:** 2013-12-05

**Authors:** Tünzale Bayramoğlu, Oğuz AKKUŞ, Kamil Nas, Miklós Illyes, Ferenc Molnar, Emel Gürkan, M. Bayram Bashırov, Şerafettin Demir, Gamze Akkuş, Esmeray Acartürk

**Affiliations:** 1 Çukurova University, Faculty of Medicine, Department of Cardiology, Adana, Turkey; 2 Szent János Hospital Department of Radiology, Budapest, Hungary; 3 Heart Institute Faculty of Medicine, University of Pécs, Pécs, Hungary; 4 Department of Hydrodynamic Systems, Budapest University of Technology and Economics, Budapest, Hungary; 5 Çukurova University, Faculty of Medicine, Department of Internal Medicine, Hematology, Adana, Turkey; 6 Çukurova University, Faculty of Medicine, Adana, Turkey; 7 Adana State Hospital, Department of Cardiology, Adana, Turkey

**Keywords:** sickle cell, Arterial stiffness, Pulse wave velocity, Quality of life

## Abstract

**Objective:** Pulse wave velocity (PWV) and aortic augmentation index (AI) are indicators of arterial stiffness. Pulse wave reflection and arterial stiffness are related to cardiovascular events and sickle cell disease. However, the effect of these parameters on the heterozygous sickle cell trait (HbAS) is unknown. The aim of this study is to evaluate the arterial stiffness and wave reflection in young adult heterozygous sickle cell carriers.

**Materials and Methods:** We enrolled 40 volunteers (20 HbAS cases, 20 hemoglobin AA [HbAA] cases) aged between 18 and 40 years. AI and PWV values were measured by arteriography.

**Results:** Aortic blood pressure, aortic AI, and brachial AI values were significantly higher in HbAS cases compared to the control group (HbAA) (p=0.033, 0.011, and 0.011, respectively). A statistically significant positive correlation was found between aortic pulse wave velocity and mean arterial pressure, age, aortic AI, brachial AI, weight, and low-density lipoprotein levels (p=0.000, 0.017, 0.000, 0.000, 0.034, and 0.05, respectively) in the whole study population. Aortic AI and age were also significantly correlated (p=0.026). In addition, a positive correlation between aortic PWV and systolic blood pressure and a positive correlation between aortic AI and mean arterial pressure (p=0.027 and 0.009, respectively) were found in HbAS individuals. Our study reveals that mean arterial pressure and heart rate are independent determinants for the aortic AI. Mean arterial pressure and age are independent determinants for aortic PWV.

**Conclusion:** Arterial stiffness measurement is an easy, cheap, and reliable method in the early diagnosis of cardiovascular disease in heterozygous sickle cell carriers. These results may depend on the amount of hemoglobin S in red blood cells. Further studies are required to investigate the blood pressure changes and its effects on arterial stiffness in order to explain the vascular aging mechanism in the HbAS trait population.

**Conflict of interest:**None declared.

## INTRODUCTION

Sickle cell disease (SCD) affects many systems as it is a chronic and hemolytic autosomal recessive disease. Atherosclerosis is a common finding in patients with sickle cell anemia [1]. Moreover, the most common cause of morbidity and mortality in these patients are ischemic complications [2]. As a consequence of atherosclerosis, arterial stiffness increases. Arterial stiffness causes a faster reflection of the forward pulse wave from bifurcation points in peripheral vessels. As a result of the new waveform, systolic blood pressure (SBP) increases, diastolic blood pressure (DBP) decreases, cardiac workload increases, and coronary perfusion falls. The role of arterial stiffness and wave reflection has been established in many studies [3,4]. In addition, the relationship between SCD and pulse wave reflection causing stroke has been demonstrated [5]. These vascular complications develop as a result of microvascular occlusion by dense and rigid sickle cells [6]. Inversely, due to lower blood pressure in the homozygous sickle cell form (HbSS), aortic pulse wave velocity (PWV) was found to be lower than in the healthy hemoglobin AA genotype (HbAA) group [7]. Pulse wave velocity (PWV) is a susceptible diagnostic element, and it is also involved in risk stratification for subclinical organ damage [8]. Based on previous studies, if the change of wave reflection and arterial stiffness are related to cardiovascular events, there is a need for more investigations within sickle cell populations. In this study we investigated the relationship between carriers of heterozygous sickle cell (HbAS) and arterial stiffness parameters. 

## MATERIALS AND METHODS

**Patients**

Twenty individuals with HbAS (16 women and 4 men, mean age of 28.65±6.50 years) and 20 healthy participants with HbAA as a control group (16 women and 4 men, mean age of 31.10±5.86 years) were included in the study. Diagnosis was made by hemoglobin electrophoresis and family screening in both groups. Atrial fibrillation and/or flutter, chronic renal failure, mild or severe valvular heart disease, and other chronic diseases were the exclusion criteria. Our local ethics committee approved the study and written informed consent was obtained from all participants. 

**Physical Examination**

Blood pressures were measured with the aid of a mercury sphygmomanometer after subjects rested for at least 15 min and had not consumed caffeinated beverages or tobacco in the last 12 h. We recorded heart rate by counting the number of heart beats in 1 min. Circulatory and cardiac examinations were performed. Skin pallor, cold extremities, peripheral cyanosis, cardiac cachexia, cardiac murmurs, increased apex beat, and third and fourth heart sounds were noted as pathological findings on physical examination. 

**Laboratory Examination**

 A 12-lead electrocardiogram was recorded on admission. Fasting venous blood samples were taken after 12 h of fasting. Complete blood count, thyroid function tests, fasting blood glucose, blood urea, creatinine, sodium, potassium, total cholesterol, low-density lipoprotein cholesterol (LDL-C), and hemoglobin electrophoresis were checked for all participants. 

**Echocardiographic Examination**

All echocardiographic measurements were obtained from the patients at rest. Standard echocardiographic examination and pulsed-wave Doppler and tissue Doppler imaging were performed on an ACUSON SequoiaTM ultrasound machine (Siemens Medical Solutions, USA) with a 2.5- or 3.5-MHz phased-array transducer. The mean of all recordings from 3 consecutive cycles was used for measurements. M-mode measurements of left ventricular end-diastolic and end-systolic dimensions and volumes, ventricular septal and posterior wall thicknesses, and left atrial end diastolic dimensions were calculated in accordance with the recommendations of the American Society of Echocardiography [9]. Left ventricular mass was calculated by use of the Penn formula (1.04 × [(SVd + IVSd + ADd)³ – (SVd)³] – 13.6). Left ventricular ejection fraction was calculated by use of the modified Simpson technique. Left ventricular diastolic function was evaluated in the apical 4-chamber view by means of pulsed-wave and tissue Doppler imaging. The pulsed-wave Doppler imaging was performed in order to measure transmitral flow values, including the peak early diastolic filling velocity (E), the peak late diastolic filling velocity (A), the early diastolic/late diastolic filling velocity (E/A) ratio, the E-wave deceleration time, and the isovolumic relaxation time. The tissue Doppler imaging was performed in order to measure systolic myocardial velocity (Sm), peak early diastolic myocardial velocity (Em), and peak late diastolic myocardial velocity (Am). The Em/Am ratio was calculated at the end of expiration. 

**Pulse Waveform Analysis**

Assessment of arterial stiffness was performed noninvasively with the commercially available Arteriograph (TensioMed, Budapest, Hungary) [10]. We measured the participants’ oscillometric pulse waves and the distance between the jugulum and symphysis (which is the same as by the invasive method for the distance between the aortic root and the aortic bifurcation), and the PWV was calculated. Pulse waves were recorded at suprasystolic pressure. The oscillation signs were identified from a cuff inflated to at least >35 mmHg above the systolic blood pressure. In this state there is complete brachial artery occlusion and it functions as a membrane before the cuff. Pulse waves hit the membrane and oscillometric waves were measured by the device, and we could see the waveforms on the monitor. The augmentation index (AI) was defined as the ratio of the difference between the second (P2, appearing because of the reflection of the first pulse wave) and first systolic peaks (P1, induced by the heart systole) to pulse pressure (PP), and it was expressed as a percentage of the ratio (AI = [P2 – P1] / PP × 100). SBP, DBP, PP, central aortic pressure (AP), heart rate, and other hemodynamic parameters were expressed as return time (RT, measured in seconds), and diastolic reflection area (DRA), systolic area index (SAI %), and diastolic area index (DAI %) were measured noninvasively with the TensioMed Arteriograph. DRA reflects the quality of the coronary arterial diastolic filling, while SAI and DAI are the areas of the systolic and diastolic portions under the pulse wave curve of a complete cardiac cycle, respectively. Hence, coronary perfusion is better when DAI and DRA values are higher. Furthermore, RT is the PWV time from the aortic root until the bifurcation and return, and so this value gets smaller as the aortic wall gets stiffer. 

**Statistical Analysis**

Statistical analysis was performed using SPSS 13.0. Categorical measures were summarized as number and percentage; numerical measures were summarized as mean and standard deviation (or, wherever necessary, median and minimum-maximum). The chi-square test was used to compare categorical measurements between the groups. The quantitative measurements of independent groups were compared by either t-test or Mann–Whitney U test for parametric and non-parametric data, respectively. Univariate analysis was used to determine the correlations between PWV and AI, SBP, heart rate, weight, height, fasting plasma glucose, serum urea, creatinine, LDL-C, total cholesterol, and Hb. Stepwise multiple regression analysis was used to determine whether HbAS, age, weight, mean arterial pressure (MAP), heart rate, Hb, and the value of total cholesterol were independent predictors of PWV and AI. The mutual relationship of PWV and AI with blood pressure and heart rate was determined by covariance analysis. In correlation analysis, p≤0.01 was considered significant. In other analyses, we treated p≤0.05 as significant. 

## RESULTS

**Figure 1:**
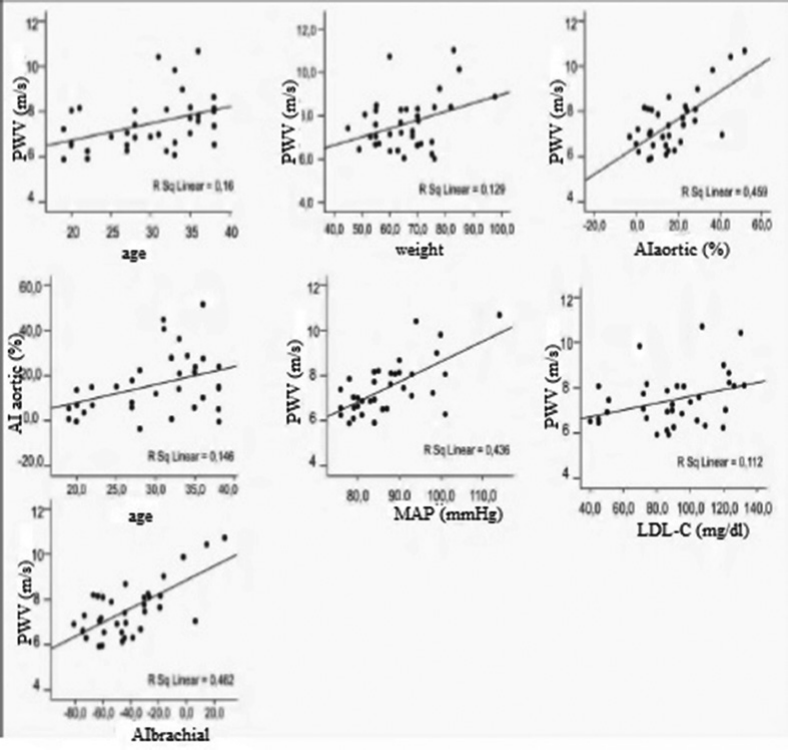
Correlations between pulse wave velocity (PWV) and mean arterial pressure (MAP), age, weight, aortic augmentation index (AI-aortic), brachial augmentation index (AI-brachial), and low-density lipoprotein cholesterol (LDL-C) in the whole study population

Table 1 shows the clinical, laboratory, and hemodynamic characteristics of the study population. Age, weight, and height were similar between groups. Aortic pressure, sodium, potassium, LDL-C, total cholesterol, mean corpuscular volume, mean corpuscular hemoglobin, and red cell distribution width were significantly different between groups. In terms of arterial stiffness parameters, only aortic AI and brachial AI were significantly higher in HbAS individuals (p=0.011). Considering the whole study population, positive correlations were found between PWV and mean arterial pressure, age, weight, aortic AI, brachial AI, and LDL-C (Figure 1). Table 2 shows the relationship between statistically significant variables in the whole study population. Furthermore, aortic AI increased with age and decreased with higher heart rate (p≤0.05). 

**Figure 2:**
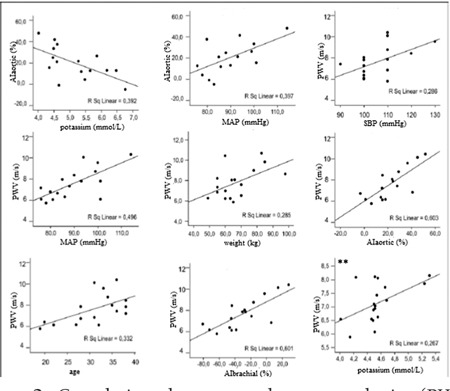
Correlations between pulse wave velocity (PWV) and aortic augmentation index (AI-aortic) and brachial augmentation index (AI-brachial), systolic blood pressure (SBP), mean arterial pressure (MAP), age, weight, and potassium in HbAS individuals

Positive correlations were found between PWV and AI and SBP, MAP, age, and weight in HbAS individuals (Figure 2). PWV increased with higher values of SBP, MAP, age, and weight (p=0.000, p=0.002, p=0.016, and p=0.027, respectively). Aortic AI and MAP were also found to have a positive correlation (p=0.009). A negative correlation was found between aortic AI and serum potassium levels (p=0.007). In contrast to aortic AI, considering all groups there was no correlation between PWV and heart rate. Serum potassium level was higher in HbAS carriers. This may have been due to more hemolysis while the blood samples of carriers were being held in the in vitro hypoxic environment. There was no statistically significant association between PWV and serum potassium concentration in sickle cell carriers. However, in the group of HbAA participants with high potassium levels, PWV was found to be increased (p=0.028). Table 3 shows the separate relationships between statistically significant variables and HbAS and HbAA by multivariate analysis.

After performing multiple stepwise regression analysis, we established that PWV and aortic AI were both independently positively associated with MAP (p=0.003 and 0.002, respectively). At the same time, PWV was also positively associated with age (p=0.029). The aortic AI was independently negatively associated with heart rate (p=0.041). Multiple stepwise regression analysis results are shown in Tables 4 and 5. 

## DISCUSSION

Cardiovascular effects of pulse reflection and arterial stiffness have been demonstrated. The effect of vascular aging on prognosis has been proven in publications about many diseases. Major determinants of these detrimental results were pulse pressure [11], AI [12], and PWV [13]. PWV is an indicator of subclinical organ damage at values higher than 12 m/s [8]. Sickle cell anemia is a hereditary disorder causing abnormal hemoglobin synthesis. Sickle cell patients (HbSS) are often admitted to the hospital because of the painful symptoms, and this process is associated with shortened life expectancy [2]. However, HbAS is a benign disorder with a standard life-span outside of cases of vigorous exercise or in military pilots [14,15]. There are many comprehensive studies related to the hemodynamic changes as a complication of the disease [16,17]. Some of these studies were associated with arterial stiffness and wave reflection [5,7,18]. 

Rees et al. [19] measured blood concentrations of nitric oxide in patients with SCD. Nitric oxide causes hypotension due to vasodilatation. Concentrations were higher than in the healthy control group. They also identified similar values during painful-crisis and steady-state SCD, but higher values than in hemoglobin E/beta-thalassemic form. HbSS patients experienced painful/hemolytic crises more often than heterozygous patients (HbAS). Lemogoum et al. [7] examined HbSS patients to investigate the connection between lower blood pressure and arterial stiffness. They excluded patients who experienced painful/hemolytic crises. SBP, DBP, and MAP were significantly lower in HbSS patients. Pulse pressure is found to be increasing as arterial stiffness worsens. Benetos et al. [11] determined the value of pulse pressure to predict cardiovascular outcomes. In our study, central aortic pressure was higher in the HbAS group (p=0.033). SBP, DBP, MAP, and pulse pressures were also higher in the HbAS group, but these results were not statistically significant. Aortic PWV was similar between HbAA and HbAS participants. Aortic and brachial AI values were significantly higher in the HbAS group (p=0.011). In the current study, blood pressure values were similar between groups, except for central aortic pressure. AI values were found to be higher in HbAS carriers. PWV was not significantly different between groups in our study, in contrast to patients with HbSS according to the findings of Lemogoum et al. [7]. In light of these results, the lower blood pressure can explain the reduced PWV in homozygous (HbSS) patients and the positive association between arterial stiffness and blood pressure. However, blood pressure and wave reflection changes may be affected by factors other than nitric oxide in homozygous and heterozygous forms of disease.

The influence of age-dependent vascular damage and the effect of arterial stiffness are well established [20]. There was no significant difference between groups in terms of mean age in this study. There was a positive association between PWV with age in the entire study population and among HbAS carriers (p=0.017 and 0.016, respectively). Demirci et al. [21] suggested that worsening arterial stiffness was the most related variable to higher MAP values. In stepwise multiple regression analysis we obtained a positive correlation between PWV and MAP (p=0.003). PWV was increased by higher MAP. Cypien et al. [22] examined MAP and arterial stiffness in women and, after multiple regression analysis, MAP was found to be associated with AI (p<0.001). For each 10% increase in AI, the risk of mortality related to coronary events was increased by 28% [23]. In our study, the most determinative predictors of arterial stiffness were aortic AI and brachial AI according to univariate analysis. These values were higher in HbAS patients. According to stepwise multiple regression analysis, there was a positive correlation between aortic AI and MAP (p=0.002). Bahl et al. [24] identified higher heart rates in patients with low hemoglobin levels (below 7 mg/dL). Nevertheless, there was no significant heart rate change in patients with HbSS, except for extreme cases (painful/hemolytic crisis) [25]. Our heterozygous patients had similar hemoglobin and hematocrit levels as those in the HbAA group. Mean corpuscular volume and mean corpuscular hemoglobin were lower in patients with HbAS (p<0.001). As a result, heart rate (beats/min) values were not distinct between groups. Wilkinson et al. [26] assessed the effects of changes in heart rate on wave reflection and arterial stiffness. AI was significantly decreased with higher heart rate and was much more sensitive to the effect of heart rate in their study. Our results were compatible with their findings, as the aortic AI was inversely related to heart rate (p=0.041).

Pannier et al. [27] examined the simultaneous PWV measurements of the aorta, brachial, and femoral arteries in 305 patients and unequivocally proved that only the PWV measurements of the aorta had a predictive value. We measured PWV only from the aorta.

Noninvasive measurement of arterial stiffness is a valuable method. Arteriography results have a considerably tight relationship with cardiac catheterization measurements [10]. Nevertheless, a study comparing other devices that can measure PWV showed that similar PWV values were obtained using the SphygmoCor (8.1±1.1 m/s) or the Arteriograph (8.6±1.3 m/s). However, for the Complior method, values were significantly different (10.1±1.7 m/s) because the recorded travel distance for PWV was higher than the others [28].

Our study revealed that MAP and heart rate were independent determinants for the aortic AI. MAP and age were also independent determinants for aortic pulse wave velocity. The most important independent predictors of arterial stiffness were MAP and age. 

## CONCLUSION

Sickle cell disease (HbSS) is associated with shortened life expectancy, although the sickle cell trait (HbAS) is a benign carrier condition and is not associated with shortened life expectancy in ordinary people. HbAS patients have a better life quality with fewer complaints than HbSS patients, but still at levels lower than among the normal healthy population. Unpredictable cardiovascular collapse and death may occur in the HbAS population during or after vigorous exercise. Therefore, measurement of arterial stiffness might help achieve a better understanding of complications associated with sickle cell carriers. Arterial stiffness measurement is an easy, cheap, and reliable method in the early diagnosis of cardiovascular disease in heterozygous sickle cell carriers. These results may depend on the amount of S hemoglobin in red blood cells. Further studies are required to investigate blood pressure changes and their effects on arterial stiffness in order to explain the vascular aging mechanism in patients with sickle cell disorder.

## CONFLICT OF INTEREST STATEMENT

The authors of this paper have no conflicts of interest, including specific financial interests, relationships, and/ or affiliations relevant to the subject matter or materials included.

## Figures and Tables

**Table 1:**
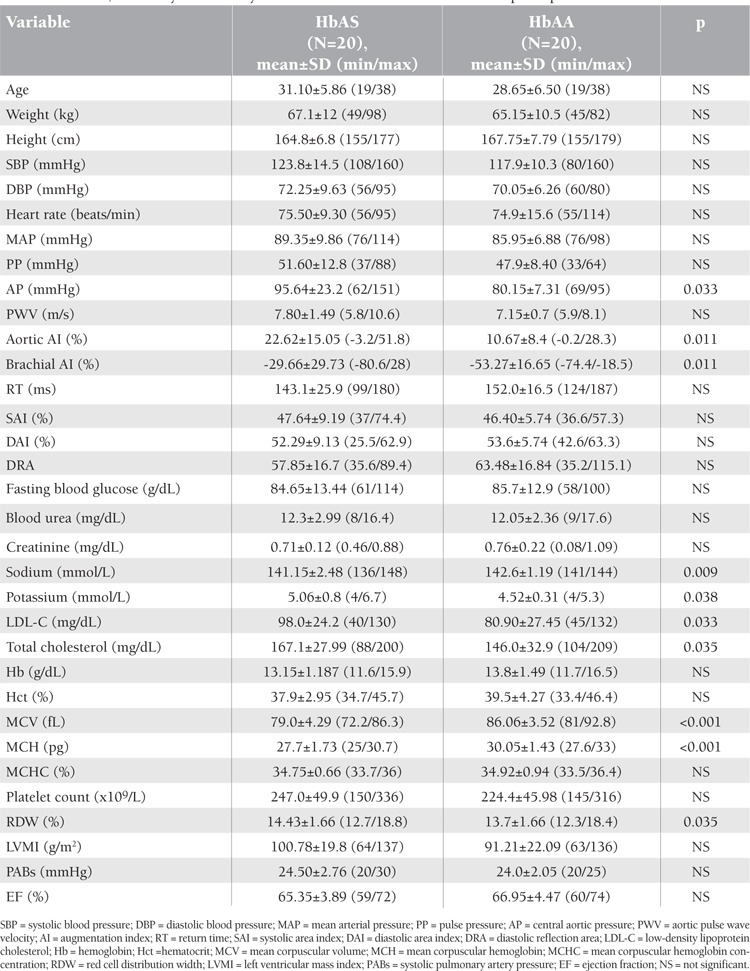
Clinical, laboratory and hemodynamic characteristics of HbAS and HbAA participants

**Table 2:**
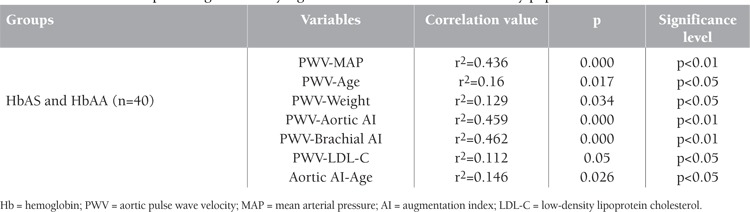
The relationships among statistically significant variables in the whole study population

**Table 3:**
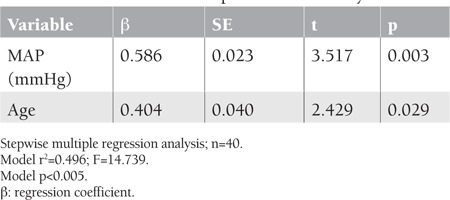
The relationships among statistically significant variables in HbAS and HbAA participants

**Table 4:**
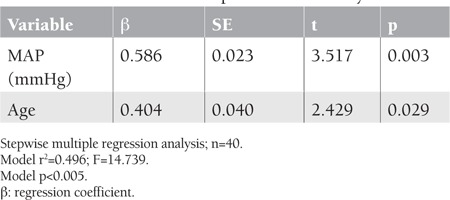
Predictors of aortic pulse wave velocity

**Table 5:**
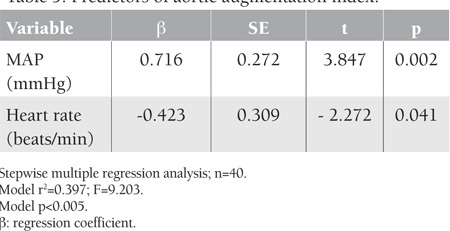
Predictors of aortic augmentation index
